# Post-Toilet Wiping Style Is Associated With the Risk of Urinary Tract Infection in Women

**DOI:** 10.7759/cureus.58107

**Published:** 2024-04-12

**Authors:** Tetsuya Akaishi

**Affiliations:** 1 Department of Education and Support for Regional Medicine, Tohoku University Hospital, Sendai, JPN

**Keywords:** post-toilet wiping style, urinary tract infection, toilet habits, risk factor, women’s health

## Abstract

Introduction: Urinary tract infection (UTI) has a lifetime incidence of ≥50% in women. A wide variety of clinical, physiological, and lifestyle risk factors for UTI have been identified, but the exact relationship between post-toilet anal and perineal hygiene practices, especially the wiping direction with toilet paper, and UTI risks has not been investigated yet. Therefore, this study cross-sectionally investigated the post-toilet wiping habit and lifetime UTI events in the general population.

Methods: Individuals who visited two hospitals in Japan between April 2020 and March 2023 were initially recruited. Self-reported questionnaires regarding post-toilet wiping habits and past UTI events were collected, and their relationship was investigated in males and females. Subgroup analyses by age were further performed to estimate the impact of age on the relationship.

Results: A total of 294 individuals (141 males and 153 females) agreed to participate and answered the question of post-toilet wiping direction. The number of individuals with post-toilet wiping with the arm from the front between the legs was 32 (23%) in males and 68 (44%) in females. The lifetime UTI events were more frequent in females than in males (p<0.0001). The impact of post-toilet wiping with the arm from the front between the legs on UTI events, adjusting for the age and history of diabetes mellitus, was not statistically significant both in males and females (p≥0.10 for both). Meanwhile, when the relationship was evaluated by different age groups, wiping habits from the front and UTI were significantly associated with each other in middle-aged women aged 40-59, whereas they were not in younger and older age groups.

Conclusion: Approximately 40-50% of women performed post-toilet wiping with the arm from the front between the legs. This post-toilet wiping habit was suggested to be a potential risk of UTI in women, especially in middle-aged subgroups, and may be better to be changed to wiping from behind.

## Introduction

Urinary tract infection (UTI) is known to have various risk factors and predisposing conditions, such as diabetes mellitus, sexual intercourse, menstrual hygiene, holding urine for a long time, dehydration, and urinary incontinence [1-6]. UTI is known to occur several times more frequently in women than in men, with a supposed lifetime incidence of more than 50% in women [7,8]. The most common isolated pathogen in women with UTI is Escherichia coli, suggesting the potential importance of post-toilet anal and perineal hygiene practices to prevent UTI [9-11]. However, the exact relationship between the post-toilet anal hygiene practices, including the wiping direction with toilet paper, currently remains unknown. Establishing such practicable post-toilet anal hygiene management to reduce UTIs will contribute to the better well-being of women in the general population. Therefore, this cross-sectional observational study investigated the relationship between the post-toilet wiping style and the lifetime incidence of UTI events among males and females in the general population.

## Materials and methods

Study design

The present study initially recruited individuals who visited the following two hospitals in Japan between April 2020 and March 2023: Kesennuma City Municipal Motoyoshi Hospital (Kesennuma, Japan) and Tohoku University Hospital (Sendai, Japan). Those who were recruited from the former visited the hospital for a regular medical check-up, and those who were recruited from the latter visited for a detailed examination with unexplained miscellaneous physical complaints and/or laboratory data abnormalities. Although it is known that UTI is not common in males with a normal anatomical urinary tract, this study enrolled both males and females to clarify the sex difference for the impact of post-toilet wiping direction on UTI events. The most common physical symptoms among those who visited Tohoku University Hospital were unexplained pain or paresthesia in the body (30-40%), followed by chronic fatigue (15-20%). Among those who were recruited, those who agreed to participate in this study and offered written informed consent were evaluated in the subsequent analyses.

Collected data

**Figure 1:**
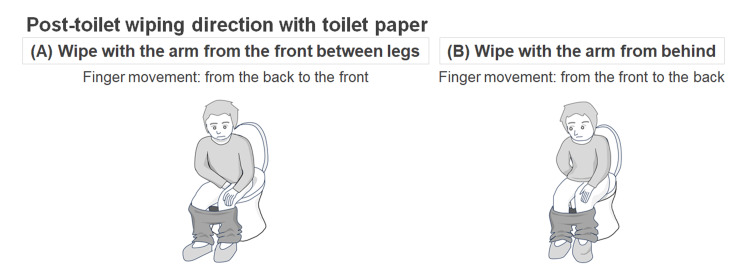
Illustrations of post-toilet wiping style Post-toilet wiping with toilet paper (A) with the arm from the front between the legs and (B) with the arm from behind is shown. The finger movements in the left panel are from the back to the front, while those in the right panel are from the front to the back.

From the participants, data regarding age, sex, direction of wiping the bottom with toilet paper after toileting, and lifetime total events of UTI were collected. As a potential risk factor for UTI, the history of diabetes mellitus (DM) was also collected. The age and lifetime UTI events were used as the quantitative variables, while the sex and post-toilet wiping direction were used as the qualitative variables. Illustrations showing the evaluated type of post-toilet wiping are shown in Figure 1. The left panel shows a wiping with the arm from the front between the legs (i.e., finger movement from the back to the front), while the right panel shows a wiping with the arm behind the bottom (i.e., finger movement from the front to the back).

Statistical analyses

The distributions of the quantitative variables were described as the median and interquartile range (IQR; 25-75 percentiles). Quantitative variables between independent groups were compared with the Mann-Whitney U test. The frequency of a specific characteristic between two groups was compared with the chi-square test or Fisher’s exact test according to the expected frequency of individuals in each subgroup. Multivariable analyses simultaneously using explanatory variables of categorical (e.g., sex, history of DM, post-toilet wiping direction) and quantitative data (e.g., age, lifetime UTI events) were performed with the analysis of covariance (ANCOVA). A p-value threshold of p<0.05 was used. All statistical analyses were performed with the R Statistical Software version 4.1.3 (R Foundation, Vienna, Austria).

Ethics

This study was approved by the Institutional Review Board of the Tohoku University Graduate School of Medicine (approval number: 20201063). Written informed consent was obtained from all participants. The study was performed in accordance with the latest version of the Declaration of Helsinki, as revised in 2013.

## Results

Participants

Among the overall 328 individuals (157 males and 171 females) who agreed to participate in this study, 294 individuals (141 males and 153 females) answered both questions of (1) post-toilet wiping direction of the anus with toilet paper and (2) total lifetime events of UTI that required oral antibiotics. The collected data by gender from the 294 individuals is summarized in Table 1. The distribution of age did not differ between the male and female participants (median 47 years [IQR 34-59 years] vs. median 48 years [IQR 34-60 years]; p=0.9245, Mann-Whitney U test). The total number of UTI events was significantly lower in males compared to females (median 0 [IQR 0-0] vs. median 0 [IQR 0-2]; p<0.0001, Mann-Whitney U test). The age-adjusted lifetime UTI events were still significantly higher in females compared to males (p<0.0001, ANCOVA). Nearly half of the women had a post-toilet wiping habit with the arm from the front between the legs, while only 20-30% of the men had this habit (p=0.0001, Fisher’s exact test).

**Table 1:** Demographics and post-toilet wiping habits among the participants by gender Distributions of age and lifetime UTI events are described as the median and interquartile range. DM: diabetes mellitus; UTI: urinary tract infection * Individuals from Tohoku University Hospital (n=167) visited the hospital for detailed examinations with unexplained miscellaneous physical symptoms or laboratory abnormalities. None of them visited the hospital with the complaint of repeated UTI events. Individuals from Motoyoshi Hospital (n=129) visited the hospital to undergo a regular medical check-up. † Lifetime number of UTI events that required oral antibiotics.

Characteristics	Males (n=141)	Females (n=153)	P
Age	47 (34–59) years	48 (34–59) years	0.9442
Tohoku University Hospital, n (%) *	75 (53%)	92 (60%)	0.2792
Motoyoshi Hospital, n (%) *	66 (47%)	61 (40%)	
History of DM, n (%)	11 (7.8%)	6 (3.9%)	0.2113
Lifetime UTI events, n^ †^	0 (0–0) times	0 (0–2) times	<0.0001
Post-toilet wiping habit in overall participants, n (%)			
Wipe with the arm from the front between legs	32 (23%)	68 (44%)	0.0001
Others (Wipe with the arm from behind)	107 (76%)	84 (55%)	
Others (Do not wipe)	2 (1.4%)	1 (0.7%)	
Post-toilet wiping habit in those aged 39 years or younger, n (%)			
All	49	55	0.1529
Wipe with the arm from the front between legs	13 (27%)	23 (42%)	
Others (Wipe with the arm from behind)	36 (73%)	32 (58%)	
Others (Do not wipe)	0 (0.0%)	0 (0.0%)	

Relationship between wiping style and UTI by gender

**Figure 2:**
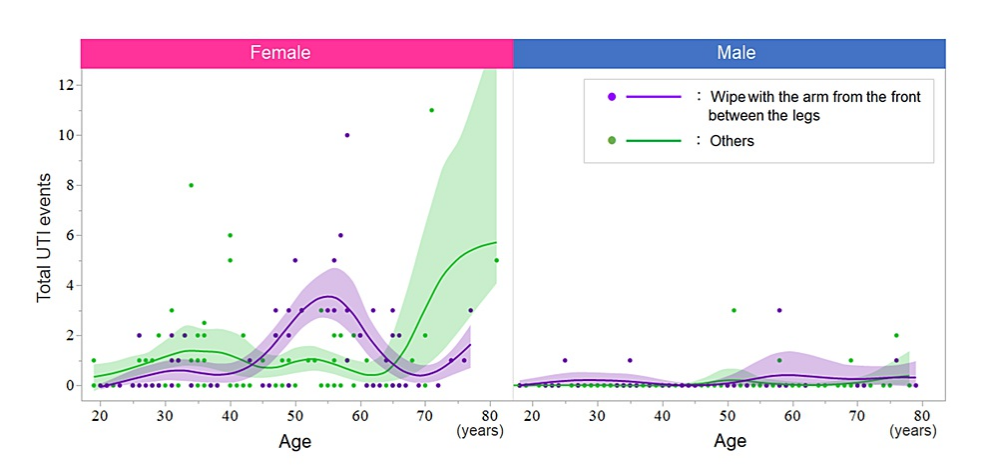
Scatterplots between age and total times of urinary tract infection by gender Post-toilet wiping style with the arm from the front between the legs was significantly associated with increased lifetime UTI events in middle-aged women, whereas it was not observed in younger-aged women or in men.

Next, the relationship between the post-toilet wiping style and the lifetime UTI events was evaluated by performing the ANCOVA on each of the male and female participants. In each sex group, the lifetime UTI event was used as the outcome variable, and the age, history of DM, and post-toilet wiping direction were used as the explanatory variables. In the male participants, the history of DM showed a significant impact on the lifetime UTI events (p=0.0096), while age (p=0.3448) and post-toilet wiping direction did not (p=0.1134). In the female participants, age (p=0.0325) and history of DM (p=0.0307) showed a significant impact on the lifetime UTI events, while the post-toilet wiping direction did not (p=0.7719). Meanwhile, when the scatterplots between age and lifetime UTI events in each gender are depicted, larger numbers of lifetime UTI events with the post-toilet wiping with the arm from the front between the legs were suggested in the middle-aged women of approximately 40-60 years old (Figure 2). Based on this finding, a further subgroup analysis with women aged 40-59 years (n=61) was performed. This time, the age-adjusted impact of post-toilet wiping style on lifetime UTI events was statistically significant (p=0.0011, ANCOVA).

## Discussion

In this study, the possible risk of post-toilet wiping of the anus with the arm from the front between the legs (i.e., finger movement from the back to the front) for the development of UTI was suggested in women, but it was not suggested in men. More specifically, elevated lifetime UTI events were observed in middle-aged women aged 40-59 years old but were not observed in younger women aged 40 years or less, suggesting the long-term effect of the wiping habit on UTI events. This study was the first to statistically demonstrate the possible relationship between post-toilet wiping style and the development of UTI. Women were more likely to have the post-toilet habit of wiping the bottom with the arm from the front between the legs compared to men. Based on the findings of this study, women with this type of habit might be better off performing post-toilet wiping with the arm from behind to decrease the risk of developing a UTI.

A finding that should be discussed includes the fact that the relationship between post-toilet wiping with the arm from the front and UTI events was only observed in middle-aged women and not in older women. Among the possible theories is a recall bias among older women, possibly forgetting that UTI events occurred more than several decades ago. Another possibility includes the potential pivotal role of menstruation underlying the UTI in this age group. Since then, many studies have demonstrated a possible association between menstrual hygiene management practices and urinary or reproductive tract infections [3,12,13]. In a previous study, approximately 40% of women with UTI self-reported that they had UTI signs after menstruation [4]. Contaminated vaginal discharges and/or the changed vaginal microbiome during the menstruation period may underlie these findings [14,15]. If these theories are correct, the post-toilet habit of wiping the bottom from behind is better to be performed both for vaginal hygiene practice during the menstruation period and anal hygiene practice after defecation to prevent UTI events.

This study had several limitations. First, this study did not evaluate the use of electric bidet seats, which have rapidly spread across the country in the last several decades. Among the enrolled participants, three individuals answered that they do not use toilet paper after defecation, suggesting that they only use the electric bidet and drying function after defecation. Moreover, the use of electric warm-water bidet toilets is supposed to increase the risk of UTI as the warm-water nozzles are often contaminated with a wide range of bacteria [16]. Future studies are better suited to incorporate the data on using electric bidet seats and adjust for their use in the development of UTIs. Another limitation was that the number of older women aged 60 years or older was small in this study. Therefore, this study could not determine the risk of wiping from the front for developing UTI in older women. Finally, this study did not collect data regarding the age of having a UTI in each individual. Therefore, the exact relationship between age and the risk of UTI in women remains uncertain.

## Conclusions

The present study demonstrated a significantly higher frequency of UTI events in women compared to men. Subgroup analyses by age demonstrated a potential risk of post-toilet wiping with the arm from the front between the legs for the development of UTI in middle-aged women aged 40-59 years old. This relationship was not observed in younger women, older women, or men. Nearly half of the women in this study performed post-toilet wiping with the arm from the front between the legs. This wiping habit could be a risk of developing a UTI, and it may be better to switch to post-toilet wiping from behind to prevent the UTI.
